# Incidence of Obstetric and Foetal Complications during Labor and Delivery at a Community Health Centre, Midwives Obstetric Unit of Durban, South Africa

**DOI:** 10.5402/2011/259308

**Published:** 2011-07-31

**Authors:** Monjurul Hoque

**Affiliations:** Kwadabeka Community Health Centre, KwaZulu-Natal, P. O. Box 2468, New Germany 3610, South Africa

## Abstract

The objectives of this retrospective cohort study were to estimate the incidence of obstetric complications during labor and delivery and their demographic predictors. A total of 2706 pregnant women were consecutively admitted to a midwife obstetric unit with labor pain between January and December 2007 constituted the sample. Among them 16% were diagnosed with obstetrical and foetal complications. The most frequently observed foetal and obstetric complications were foetal distress (35.5/1000) and poor progress of labor (28.3/1000), respectively. Primigravid and grandmultiparity women were 12 (OR = 11.89) and 5 (OR = 4.575) times, respectively, more likely to have complications during labor and delivery. Women without antenatal care had doubled (OR = 1.815, 95% CI, 1.310; 2.515) the chance of having complications. Mothers age <20 years was protective (OR = 0.579, 95% CI, 0.348; 0.963) of complications during delivery compared to women who were ≥35 years. National and local policies and intervention programmes must address the need of the risk groups of pregnant women during labor and delivery.

## 1. Introduction

Obstetric and foetal complication and their management have effects on a pregnant mother or her foetus. Pregnant women experience these complications during pregnancy, delivery, and up to 42 days following childbirth. An estimated 15% of pregnant women in developing countries experience pregnancy-related complications and nearly 530,000 women worldwide die annually with 95% of these deaths occurring in Africa and Asia [1, 2]. Between two and three million African women are also found handicapped from obstetric complications each year [3, 4]. According to the World Health Organization (WHO), reproductive health problems account for more than one third of the total burden of diseases in women [5]. Reducing maternal and child morbidity and mortality has been enshrined as one of the Millennium Development Goals (MDGs). Yet of all the health-related MDGs, there has been little progress made towards these goals [6].

The estimated maternal mortality ratio of South Africa (SA) is estimated at 147 per 100,000 live births for the year 2004 and it is higher (154) for KwaZulu-Natal province (KZN) [7]. Additionally, another 32,000 to 111,000 SA women suffer from morbidities/disabilities caused by complications during pregnancy and delivery [8]. The main causes are well known: dystortia and uterine rupture, haemorrhage, infection, hypertensive disorders of pregnancy, embolism, and anaesthesia-related complications [9]. Medical audits from SA and other parts of the world on maternal deaths revealed that more than 80% of these deaths and disabilities are preventable and that they depend on the quality of care during labor and delivery, including identification of problems, care and appropriate referrals to health facilities [7, 10–14]. As often most obstetric complications cannot be predicted or prevented during pregnancy, thus diagnosis and appropriate interventions during labor and delivery are essential [8, 15]. 

Until recently, the only national outcome indicator of maternal health is maternal mortality ratio [16]. However, if priorities are to be established and effective interventions to be designed to improve maternal and foetal health, the burden of morbidity among women giving birth must be defined, estimated and risk factors must be identified. The Healthy People 2010 and Safe Motherhood initiative objectives include new maternal health indicators including maternal morbidity during labor and delivery [16–18]. Several studies have shown that pregnancy complications during pregnancy are common and estimated between 15 and 25 for every 100 deliveries in USA [19–22]. However, most maternal deaths and serious complications occur during the time of labor and delivery [23]. Thus measuring the incidence of complications is essential for the specific population or at a health facility for strengthening the service provisions (training and recruiting of staff, purchasing of equipment, etc.).

Early detection of complications of labor and delivery, and interventions including referral to higher levels of care are seen to reduce maternal and perinatal morbidity and mortality (e.g., up to one-third of neonatal deaths) in some developing countries [24, 25]. Primary prevention is also possible for some of these complications, including certain causes of haemorrhage, infection, and complications of obstructed labor. In the case of complication that cannot be prevented, the goal is to manage appropriately to prevent them from becoming severe or life threatening. Few studies have specifically examined the incidence of complications during labor and delivery in SA and in particular to KZN. The objectives of the study are to describe the demography of the pregnant population and estimate the incidence of obstetric and foetal complications during labor and delivery, and their demographic predictors.

## 2. Method

### 2.1. Setting and Population

Kwadabeka community health centre (KCHC) is a primary health care (PHC) facility for the people living in the communities of Kwadabeka and Clermont, the residence of over 150,000 black people. These communities are located within the municipal boundaries of eThekwini (Durban). Most of the dwellers are poor, unemployed, living in formal and informal type of dwelling and having well-built cultural bond with rural people of KZN and Eastern Cape Provinces. They are essentially reliant on public health services at KCHC. Maternity service at KCHC is available 24 hours a day and is run by trained and skilled midwives (midwife obstetric unit MOU). According to the national guidelines, the unit is responsible for making antenatal care available to all pregnant women, treatment of pregnancy-related common problems, management of labor and delivery services, postnatal check-ups, and management of emergencies during antenatal and delivery services as well as appropriate referral to appropriate hospitals. Three midwives during the day time (7 am to 4 pm) and 2 midwives after hours (4 pm to 7 am) are employed together with other support staffs to carry out deliveries and care for mothers and newborns. Antenatal care and delivery services are rendered at KCHC according to the national protocol and guidelines developed and implemented since 2002 [26].

### 2.2. Labor Ward Practices

When a woman attends KCHC labor ward and complains of labor pain, a proper history is taken and examinations and investigations are conducted to diagnose the problems plus assess the risks of pregnancy and delivery. She is then admitted to the labor ward for observations on the progress of labor. If a woman does not progress to labor in 6 hours, she is then discharged home with a diagnosis and treatment (e.g., false labor pain or other appropriate condition). Labor is diagnosed if there are painful uterine contractions accompanied by cervical effacement and dilatation and or rupture of the membrane and or presence of show. Women with active labor are then allowed to continue to deliver at KCHC using partogram (entering all observations, fluids intake and output and medications on the partogram). Alert and action lines on the partogram together with other observations (e.g., foetal heart rate, temperature of mother, BP, etc.) are used to identify complications during labor. In cases of complications or risk factors (e.g., raised BP, foetal distress, etc.) identified based on national guidelines during different stages of labor or delivery, telephonic presentation and discussion with the medical practitioner at the referring hospital is carried out by midwives. This facility does not undertake any instrumental (e.g., forcef) or operational deliveries (e.g., Caesarean section). The mother is then transported to the hospital using Emergency Medical Rescue Service's ambulance. For those deliver at KCHC, observations are done for 24 hours. Thereafter, mothers and the newborns without complications are discharged home.

### 2.3. Definition of Terms

Obstetric complications during labor and delivery are defined as a condition that adversely affects women and their foetal health during delivery. Conditions that are managed adequately without any substantial effect on the woman and the foetal health are not classified as instances of obstetrical complication. For example, first and second-degree vaginal lacerations (considered normal occurrences during delivery) and episiotomies are some of them. Existing medical conditions were also identified and classified in this study.

Foetal distress is diagnosed through foetal monitoring in the way of listening to the foetal heart with fetoscope or using cardiotocography (CTG). If the CTG trace is flat with no beat to beat variations during uterine contractions or showing deceleration of foetal heart beat or if the woman drains maconium stained liquor grade II-III this then constitutes foetal distress.

Prolonged rupture of membrane is considered if a woman experiences loss of water (liquor or Mutondi Futne) using pad checks and onset of labor pain for more than 24 hours. Poor progress of labor is diagnosed if it takes more than 8 hours in latent phase of labor without any other abnormalities (e.g., abruption placenta, UTI, or false labor). Poor progress in active phase is diagnosed if labor is prolonged and cervical dilatation is at a rate of less than 1 cm/hour (crossed alert line in partogram) and other abnormalities are excluded such as cephalo-pelvic disproportion (CPD) measured by grading of moulding and formation of caput on foetal head with no descent of foetal head but foetal presentation and condition are found normal. Poor progress in the second phase of labor is diagnosed using the measure of the foetal head descend onto the pelvic floor after 2 hours of full dilatation. Abnormal presentation is considered when the presenting part of the foetus is other than the vertex, for example, face, hand, shoulder, leg, or breech. Primary postpartum haemorrhage (PPH): excessive vaginal bleeding after delivery on the first day and the mother becomes hypothermic (<35°C) or unconscious. Retained placenta is considered when the placenta does not detach or come out for more than one hour after the birth of the baby. Grandmultiparity is considered if the pregnant mother had previous ≥5 births.

### 2.4. Study Design, Sample Selection, and Data Collection

A retrospective cohort study was accomplished aiming at all pregnant women who attended KCHC with labor pain and admitted to labor ward between January to December 2007. The number of delivery for a period of one calendar year was considered sufficient for estimating the objectives and found convenient for the study. Data were collected from labor ward admission registry. The register is the only official register of all admissions, delivery, referrals, and discharges. The register contains the name, age, parity, number of antenatal visits, complications (diagnosis) during labor, delivery, and the name of referral hospitals. Permission was sought from KCHC management team and institutional ethics review committee. No name of any patient was used in presenting data.

### 2.5. Data Analysis

Data were entered into a Microsoft Excel 2003 spreadsheet and imported to SPSS 12.0.1 for window version for analysis. The demographics and baseline outcome variables were summarized using descriptive summary measures: expressed as mean (standard deviation) continuous variables and percent for categorical variables. All statistical tests were performed using two-sided tests at the 0.05 level of significance. For all regression models, the results were expressed as effect (adjusted odds ratios for binary outcomes), corresponding two-sided 95% confidence intervals (95% CI), and associated *P* values. Both obstetrical and foetal complications were combined into one outcome for this study.

## 3. Results

A total of 3029 pregnant mothers were admitted in labor ward during the study period, 245 (8%) of them were discharged after 6 hours of observation owing to false labor or other pregnancy-related conditions (not related to labor). Another 78 (2.8%) women in labor were referred to hospitals at the time of admission for obstetric and nonobstetrical risk factors (e.g., preexisting medical conditions). Thus a total of 2706 pregnant women went into labor at KCHC and their obstetric and foetal outcomes were measured. Of these 2706 pregnant women, 430 (16%) were diagnosed with obstetrical and foetal complications during labor and delivery, and 13 (0.5%) during postdelivery period and accordingly all (443) were referred to hospitals.

### 3.1. Demographic Profile of the Pregnant Population

Teenage (age <18 years) pregnancy accounted for 15%. The mean and median ages of the sample were 24 years (SD = 5.6); majority mainstream pregnant women (82%) were below the age of 30 years (Table 1). Primigravida (nil parity) and grandmultiparity (parity > 5) accounted for 33% and 1% of all pregnant women, respectively. A higher proportion of pregnant women (92%) had received antenatal care and their mean antenatal visit was 4.

### 3.2. Obstetric and Foetal Complications

The incidences (per 1000 pregnant women) and proportions of complications during labor and delivery are shown in Table 2. The incidences of maternal and foetal complications were 12% and 4%, respectively. The most frequently observed incidence of complication was foetal distress (36.5/1000), followed by (maternal) poor progress (29.1/1000) of labor (prolong latent or active phase) and preterm labor accounted for 25.1/1000. A few (13) women developed complications (mainly PPH) after delivery and thus all complicated cases were referred to hospitals. Some women had more than one complication. Nevertheless, the women were counted only once, regardless of how many obstetric complications, preexisting medical conditions, and so forth they may have had. The results of logistic regression output for the incidence of delivery complications (both maternal and foetal) are shown in Table 3. We found that primigravid (parity nil) women were 12 times more likely (OR = 11.89, 95% CI, 1.153; 122.693) to have complication during labor and delivery. Similarly, grandmultiparity women were five times more likely (OR = 4.575, 95% CI, 1.810; 11.565) to have complication during delivery. Women who did not have any antenatal visit prior to delivery had doubled (OR = 1.815, 95% CI, 1.310; 2.515) the chance of having complications during labor and delivery compared to those who had 4 visits or more. The age of pregnant women less than 20 years had notably less chance (OR = 0.579, 95% CI, 0.348; 0.963) of developing complications compared to women who were ≥35 years.

## 4. Discussion

The present study is the pioneer, to the best of our knowledge, to estimate the incidence of maternal and foetal complications during labor and delivery at the lowest level of maternity service delivery in SA. The results confirm that the magnitude of the problem is greater than generally believed. Although the incidence of any specific type of morbidity during labor and delivery is low, the burden of total morbidity is high and the consequences thereof are grave if proper interventions are not taken. A total of 16% of pregnant women experienced any type of obstetric and foetal complications during labor and delivery and thus were referred to higher level of maternal health service facilities. This rate is higher than the rates known for Guatemala but lower than the rates observed in USA [27, 28]. In Guatemala, almost 9% of women reported complications during the antenatal period, 8% during delivery, and 4% during the postpartum period [27]. However, the postdelivery complication rate in our study is low (<1%). This low rate of postpartum complication could be due to low risk group of pregnant population of our study as the complicated cases are referred for hospitals delivery.

Foetal distress (22%), hypertensive disorders (13%), and CPD (11%) are found in higher proportions. These conditions are considered obstetric emergencies and thus required immediate/urgent and appropriate interventions. MOU unit thus requires referring these cases to a hospital for the sake of mother and the unborn baby. Prompt and effective management of complicated pregnancies and labor are now seen as central focus to reduce maternal and foetal mortality in developing countries. Staff training and transport facilities are thus important provisions to be made by the unit in charge of maternity unit of KCHC. Before transporting the complicated pregnant mothers to hospital, resuscitation of mother (intravenous fluid, oxygen inhalation, etc.) and unborn baby (intrapartum resuscitation of the baby) are crucial. Thus, regular in-service training and updates of midwives on management of obstetric complications are of paramount important. Weekly/biweekly perinatal morbidity and mortality meetings are some of the initiatives to be considered in this regards. Monthly or bimonthly feedback meetings with the referral hospital staff can also be useful to improve knowledge and skills at this lowest level of maternity service providing health facilities which are presently lacking.

Other types of complications found in this study are life threatening, and many are preventable (e.g., third-degree perineal tear, retained products, etc.). Primary prevention is possible for some of these complications, including certain causes of haemorrhage, infection, and complications of obstructed labor. Thus maternal complications during labor and delivery are a public health and clinical practice issue affecting significantly large number of women in SA. It can have an impact on foetal and infant health and can lead to maternal and foetal deaths. Though, some other serious forms of obstetric morbidity (APH, eclampsia/PET, etc.) reported in a small percentage (<3%) and thus seen to affect thousands of women each year because there are millions of deliveries annually. For example, APH was found in only 0.6% of women during childbirth among these pregnant women. Nevertheless, this life-threatening condition adds up to 13% of maternal deaths in SA [7]. Similarly, PPH was diagnosed in <3% of women delivered at KCHC. This life-threatening condition accounts for higher rates (12.4%) of maternal deaths in SA [7]. 

Measuring morbidity during labor and delivery has advantages over the previously used indicator of antepartum complications and hospitalizations [16]. Multiple admissions for certain conditions may occur, further complicating interpretation of antepartum hospitalization rates. In contrast, morbidity during labor and delivery involves hospitalizations not associated with other conditions. Moreover, the percentage of women attend health institutions for delivery has achieved at higher rates in SA (89%) and the trend is on rise [8]. A complete picture of the burden of maternal morbidity would take account of antepartum and postpartum complications. In the case of conditions/complications that occur only during childbirth or that which do not resolve until after childbirth, the incidence rates reported here accurately reflect rates during labor and delivery (e.g., obstetric trauma, pre-eclampsia, eclampsia, CPD, etc.). Conditions that occur and resolve during antepartum period and that began after 6 hours of delivery are not estimated with this methodology (e.g., antepartum infections, mastitis, etc.). 

The teenage pregnancy rate of 15% is comparable with other studies conducted in KZN [10, 29]. However, the pregnancy rate of primigravida (33%) is also similar to other studies [8, 29]. Antenatal booking rate among these pregnant women is also comparable (92%) to the rates reported in SADHS 1998 (94%) and 2003 (92%). SADHS 2003 also reported a similar rate of median antenatal visits of 4 [8, 29]. The mean antenatal visit of this population is lower. This is because of low risk group of pregnant women. We expect higher number of follow-up visits for pregnant women with complications during pregnancy. Furthermore, this study shows that primigravid and grandmultiparity women were 12 and 4.5 times more likely to have complications during delivery than those who had fewer pregnancies. Number of antenatal visits was also a vital predictor for delivery complications. This connoted those women who did not have any antenatal visits, experienced delivery complications that were 1.8 times more than those who had four or more antenatal visits. Therefore, this finding concurs with the finding of demographic risk factors for obstetric complications such as parity and antenatal visits [29–31].

Facility-based study may have limited complete population representation. As we can assume that all pregnant women from these communities do not attend KCHC or those attended other health facility (private or traditional birth attenders) during labor are not part of the study. However, it is known that nearly 90% of pregnant women in SA attend health facility for delivery and the majority (80%) attend public health facilities [32]. KCHC is situated in the heart of the community. The communities are poor and maternity service is provided free of cost. It is thus expected that most pregnant women from these communities attend KCHC for antenatal and delivery services thus represented the population. Some women had more than one obstetric complication or preexisting medical condition. Nevertheless, the women were counted only once, regardless of how many obstetric complications, preexisting medical conditions, and so forth they may have had. Pregnant women who are classified as high-risk pregnancies and are followed at hospitals for antenatal care and delivery are not part of this study thus influenced our results (underestimated the problem). Retrospective record review limited the study variables to measure different indicators. Even though the definitions of different morbidity obviously depend on the level of health services available, and the most important being the midwives' level of knowledge and experience, a particular definition and protocol is followed, and thus the identification of such conditions could be minimally deviated. International Classification of Diseases (ICD-10) codes are also not used. However, it did not misclassify the delivery conditions in our study. The preexisting medical conditions are not included as it was unclear whether these conditions were facilitated to the development of obstetric complications during labor and delivery or not. Two separate models could have been developed for two outcome variables, for example, obstetric and foetal complications. However, combining these complications as one outcome to develop the model to identify the risk groups for interventions required at the time of labor and delivery was thus considered appropriate.

## 5. Conclusion

The burden of obstetric and foetal complications during labor and delivery is high. Many of the serious types of complications identified here are preventable. If the objectives of safe motherhood and MDGs are to be achieved, national and local policies must address women's needs during labor and delivery and to meet the gaps in prevention and research programmes. Consistent and improved monitoring and appropriate intervention for maternal and foetal complications of the risk groups of pregnant women during labor and delivery are key to reduce maternal and foetal morbidity and mortality.

## Figures and Tables

**Table 1:** Demographic information of 2706 pregnant women who went into labor at KCHC during January to December 2007.

Variables	Percentage
Age in years	*n* = 2706
<18 years	14.9
19–23 years	37.8
24–28 years	25.2
29–33 years	14.4
34–38 years	6.4
39 years or more	1.2
Parity	
Nil	33.2
1–3	61.8
4–6	4.7
7 or more	0.3
No of antenatal visits	
none	8.3
1	8.0
2-3	21.7
4 or more	61.9

**Table 2:** Obstetric complication rates of 2708 pregnant women during labor, delivery, and 6 hours after delivery during January to December 2007.

Obstetric and delivery complications	Numbers in each category of complication	Proportion of all complications in percentage (*n* = 443)	Incidence (per 1000 pregnant women) of complications (*n* = 2706)
Maternal complications			

Poor progress (poor progress, prolonged latent phase)	79	17.83	29.1
Pre-term labor	68	15.35	25.1
Hypertensive disorder (eclampsia, PET, PIH)	58	13.09	21.4
CPD (CPD, labor obstruction, obstructed labor)	48	10.83	17.2
APH	17	3.83	6.4
Retained products	10	2.25	3.7
Third degree perineal tear	3	0.67	1.0
Cord prolapse	3	0.67	1.0
Other conditions	31	7.0	12.1

Foetal complications			

Foetal distress	99	22.34	36.5
Breech presentation	12	2.70	4.4
Postdelivery conditions (PPH)	13	2.89	4.8

**Table 3:** Logistic regression output for labor and delivery complications.

Variables	Adjusted OR	95% CI for OR (Lower; Upper)	Sig.
No of antenatal visit			
No visit	1.815	(1.310; 2.515)	.000
Visit one time	1.246	(0.866; 1.792)	.236
Visit 2-3 times	.867	(0.666; 1.129)	.290
Age group			
Age less than 20 years	.579	(0.348; 0.963)	.035
Age between 20–34 yrs	.645	(0.406; 1.025)	.064
Parity group			
Parity nil	11.895	(1.153; 122.693)	.038
Parity 1–3	5.079	(0.496; 51.957)	.171
Parity 4-5	3.124	(0.313; 31.181)	.332
Grandmultiparity	4.575	(1.810; 11.565)	.001
Constant	.046		.010

Variable(s) entered on step 1: grandmultiparity, number of antenatal visits (4 times or more as reference group), age group (age 20–35 years as reference group), and parity group (parity 1–5 as reference group).
